# Rapid Early Motor Improvement After Full Endoscopic Interlaminar Discectomy for L4–L5 Disc Herniation Presenting With Foot Drop: A Case Report

**DOI:** 10.7759/cureus.107214

**Published:** 2026-04-17

**Authors:** Mustafa Kaya, Muhammed Ömer Bakac, Tibet Kacira, Sengül Kocer

**Affiliations:** 1 Department of Neurosurgery, Sakarya University Faculty of Medicine, Sakarya, TUR

**Keywords:** acute motor deficit, early surgical decompression, foot drop, full-endoscopic lumbar discectomy, interlaminar approach, lumbar disc herniation, minimal invasive spine surgery

## Abstract

Lumbar disc herniation with foot drop is a clinically significant presentation because delayed decompression may adversely affect neurological recovery. Full endoscopic interlaminar lumbar discectomy (FELD-IL) is an increasingly adopted minimally invasive technique, but its role in patients presenting with acute motor deficit remains less clearly defined than in routine radiculopathy. We report a case of L4-L5 disc herniation presenting with foot drop that was managed with urgent FELD-IL and resulted in favorable early motor improvement. A 26-year-old man presented with a four-month history of low back pain radiating to the right leg, followed by three days of sudden right foot drop. Neurological examination showed weakness of ankle dorsiflexion and great toe extension (Medical Research Council grade 2/5), decreased pinprick sensation over the dorsum of the right foot and great toe, and a positive straight leg raise test. Lumbar magnetic resonance imaging demonstrated a right paracentral L4-L5 disc herniation with inferior migration compressing the traversing right L5 nerve root. The patient underwent urgent full endoscopic interlaminar lumbar discectomy under general anesthesia. Radicular pain improved immediately after surgery. On postoperative day 1, ankle dorsiflexion improved to MRC 4/5, and at two-week follow-up, the patient had a normal gait and MRC 5/5 strength on bedside examination. Early postoperative magnetic resonance imaging demonstrated satisfactory decompression of the right L5 nerve root. In this selected patient, urgent FELD-IL was technically feasible and was followed by favorable early motor improvement. This case supports the potential applicability of the interlaminar endoscopic approach in carefully selected patients with lumbar disc herniation and foot drop, but it does not establish superiority, safety, or effectiveness relative to other decompressive techniques. Larger comparative studies with longer follow-up are needed before stronger conclusions can be drawn.

## Introduction

Lumbar disc herniation is a common cause of back and leg pain in young and middle-aged adults and is traditionally treated with open or microscopic discectomy in patients who do not benefit from conservative treatment [1]. Full endoscopic lumbar discectomy (FELD) has emerged as a minimally invasive alternative that aims to reduce muscle trauma, blood loss, and hospital stay while maintaining equivalent clinical outcomes [2].

Among endoscopic techniques, the interlaminar approach is particularly suitable for L4-L5 and L5-S1, as the interlaminar window is relatively wide in these regions and the iliac wing may limit the transforaminal approach. Choi et al. first described percutaneous endoscopic interlaminar discectomy (PEID) for L5-S1 intracanalicular herniations using a rigid working channel endoscope and reported favorable clinical outcomes [3]. Subsequent series, prospective studies, and narrative reviews have confirmed that interlaminar fully endoscopic lumbar discectomy (IELD/FELD-IL) has been reported as a feasible and increasingly adopted technique in appropriately selected patients [4].

Lumbar disc herniation with acute motor deficit is clinically more consequential than pain-dominant radiculopathy because delayed decompression may adversely affect neurological recovery. While full endoscopic interlaminar lumbar discectomy (FELD-IL) is increasingly used for selected lumbar disc herniations, its specific role in patients presenting with foot drop remains less clearly defined than its role in routine radiculopathy. Here, we present a patient with L4-L5 disc herniation and foot drop who underwent urgent FELD-IL and demonstrated favorable early motor improvement. The purpose of this report is not to establish superiority of the endoscopic approach, but to illustrate its technical feasibility and early clinical outcome in a carefully selected case.

## Case presentation

A 26-year-old man presented in April 2025 with a four-month history of low back pain radiating to the right leg, predominantly over the dorsum of the foot and great toe. The pain was burning and electric shock-like, worsened by standing and walking, and had limited prolonged ambulation. During the symptomatic period, he had used oral analgesic/anti-inflammatory medication and activity modification with insufficient relief. Three days before admission, he noticed sudden weakness in right ankle dorsiflexion with tripping while walking. There was no history of trauma, prior lumbar surgery, infection, malignancy, or bowel/bladder dysfunction.

**Figure 1:**
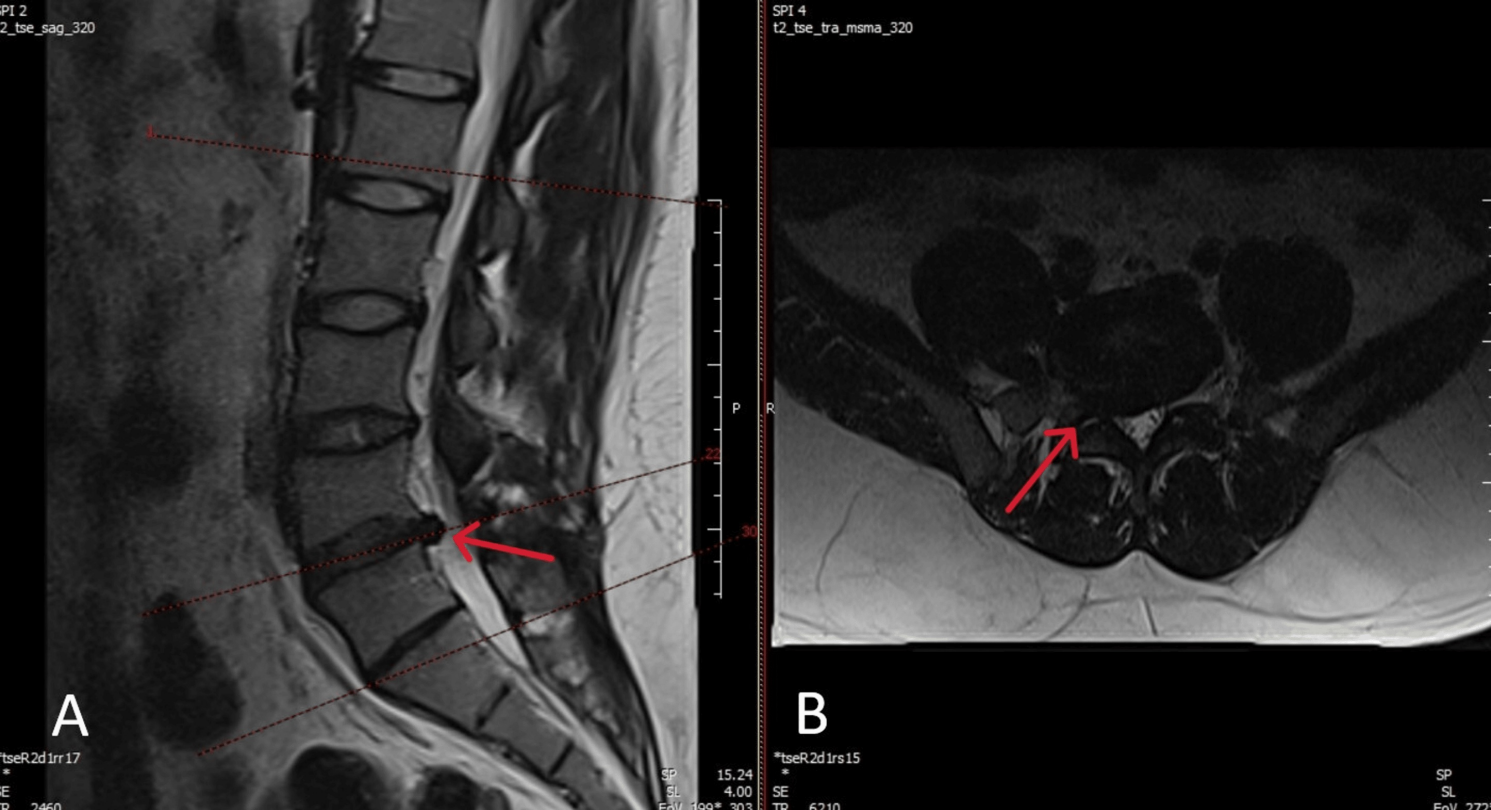
Preoperative lumbar MRI. (A) Sagittal T2-weighted MRI demonstrating L4–L5 disc extrusion (red arrow). (B) Axial T2-weighted MRI showing right paracentral disc herniation compressing the traversing right L5 nerve root (red arrow).

On examination, the straight leg raise was positive at 35° on the right. Pinprick sensation was decreased over the dorsum of the right foot and great toe. Strength of right ankle dorsiflexion and great toe extension was 2/5 on the Medical Research Council (MRC) scale, whereas plantar flexion was preserved. Deep tendon reflexes were symmetric. Lumbar MRI demonstrated a right paracentral L4-L5 disc herniation with inferior migration causing compression of the traversing right L5 nerve root (Figure 1A-1B). Because of the recent onset of motor deficit in the setting of persistent radicular symptoms and concordant MRI findings, urgent surgical decompression was recommended. In view of the favorable interlaminar anatomy at L4-L5 and the surgeon’s experience with the technique, a full endoscopic interlaminar approach was selected.

The patient underwent urgent full endoscopic interlaminar lumbar discectomy under general anesthesia in the prone position. Fluoroscopy was used for level confirmation before the skin incision. A full endoscopic interlaminar approach was then performed at the L4-L5 level through a small posterior incision. After sequential soft tissue dilation, the working cannula was introduced into the interlaminar window under fluoroscopic guidance. Continuous saline irrigation was maintained throughout the procedure to optimize visualization and facilitate safe dissection. Under endoscopic visualization, the ligamentum flavum was opened and the epidural space was accessed. The traversing right L5 nerve root and the herniated disc fragment were identified, and careful discectomy was performed until satisfactory neural decompression was achieved. No notable intraoperative bleeding or technical difficulty was encountered. Total operative time was approximately 50 minutes. The procedure was completed without complication, and the patient was transferred to recovery in stable condition.

**Figure 2:**
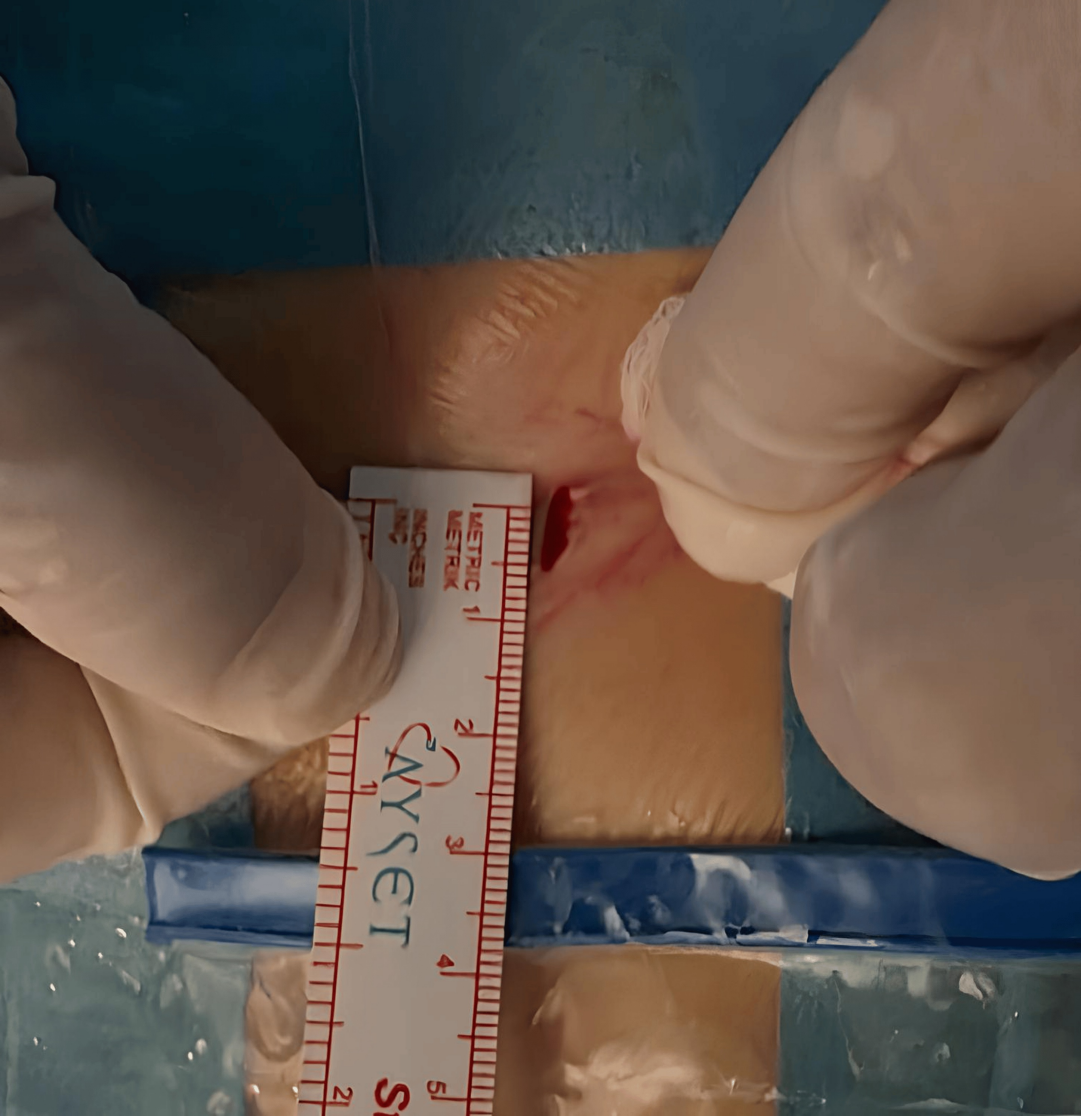
Skin incision. Small midline-adjacent skin incision (approximately 5 mm) used for the interlaminar endoscopic approach.

**Figure 3:**
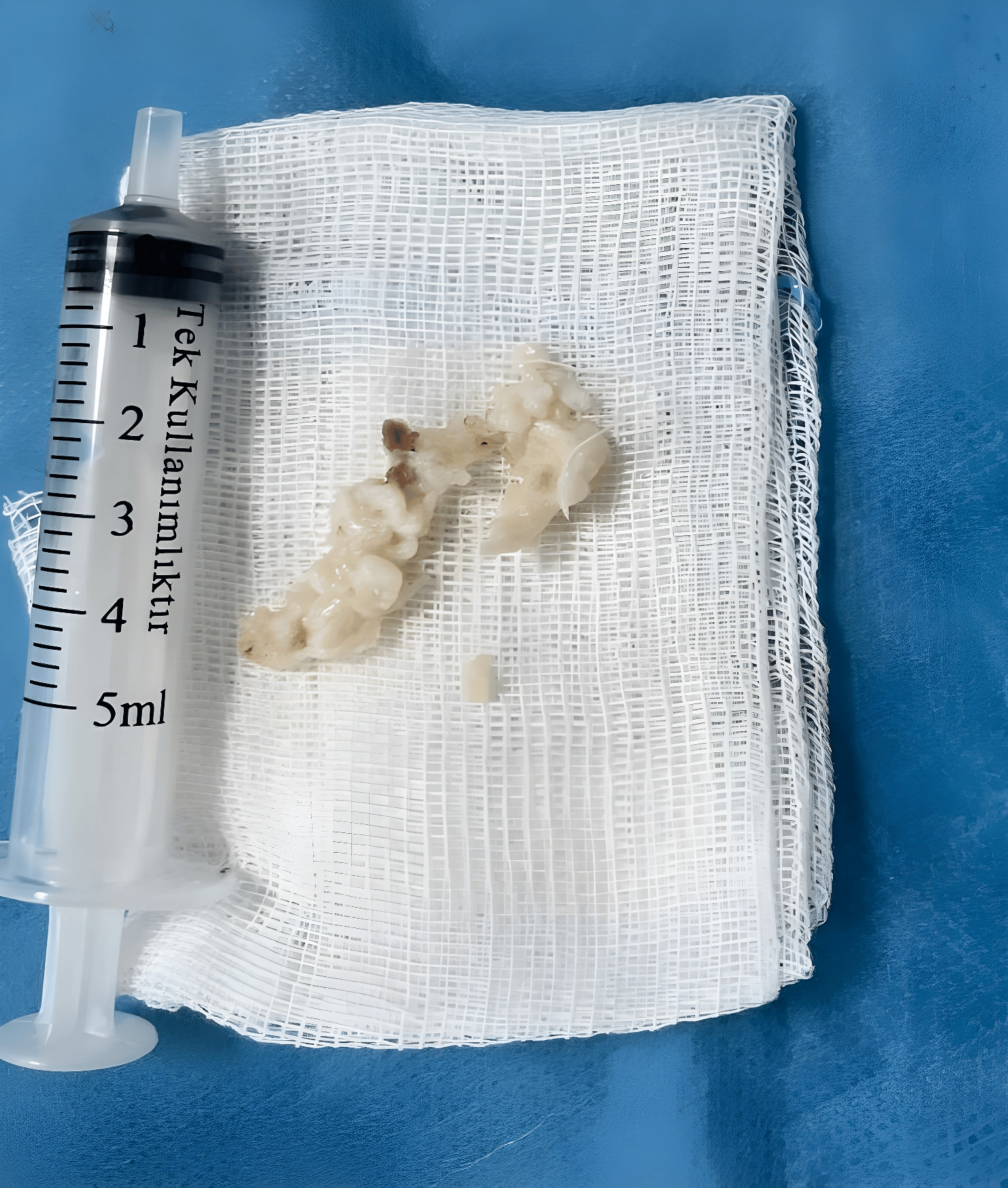
Removed disc fragments. Extruded nucleus pulposus fragments removed during the procedure.

The incision line and the removed disc material are shown in Figures 2-3. A shortened author-generated intraoperative video was provided as supplementary material via YouTube in accordance with the journal’s file size limitations and video submission format (Video 1).

**Video 1:** The intraoperative endoscopic procedure

**Figure 4:**
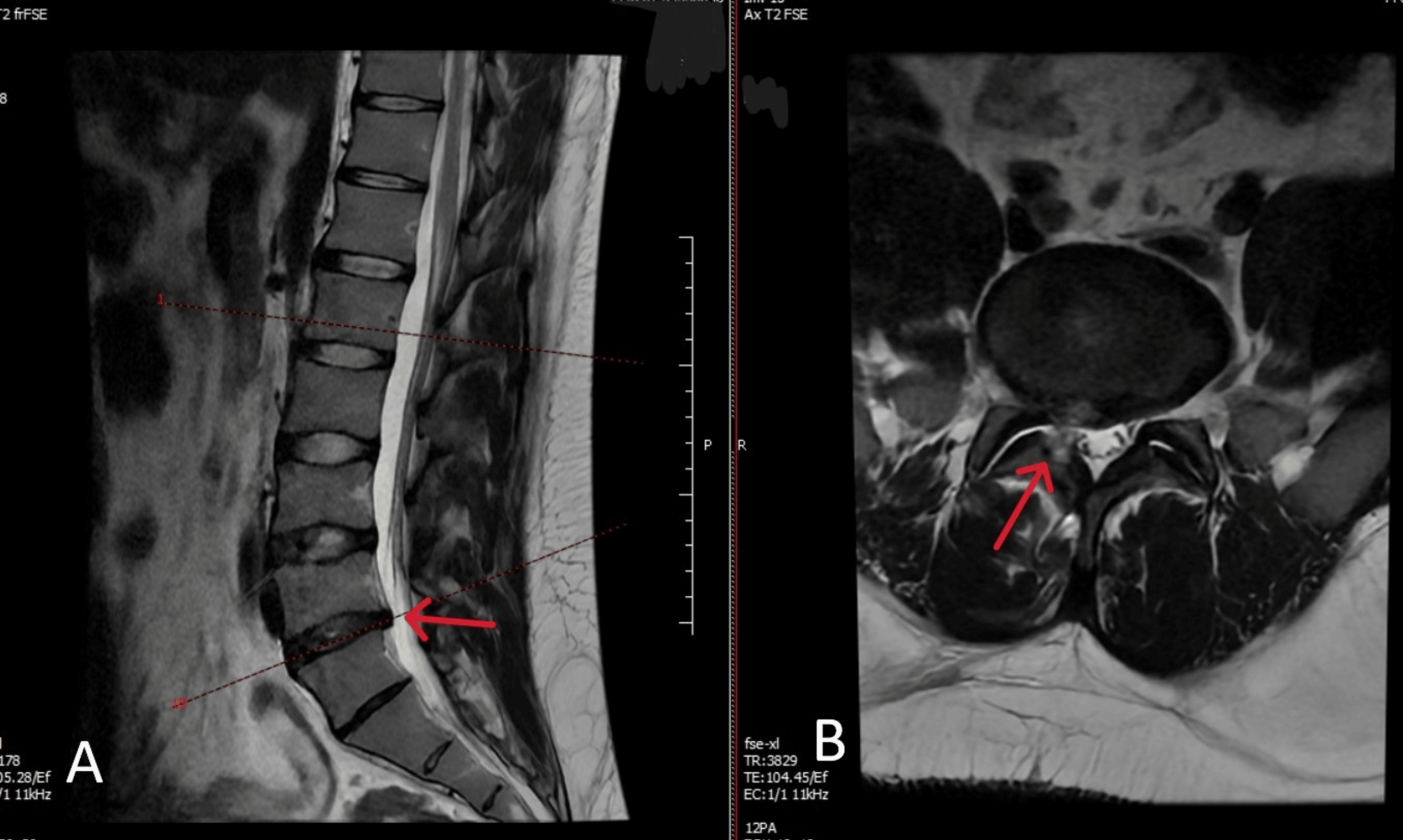
Early postoperative lumbar MRI. (A) Sagittal T2-weighted MRI showing adequate decompression at L4–L5 (red arrow). (B) Axial T2-weighted MRI demonstrating complete removal of the herniated fragment and relief of right L5 nerve root compression (red arrow).

No new neurological deficit was noted after recovery from anesthesia. Radicular pain improved immediately after surgery. On postoperative day 1, right ankle dorsiflexion improved from MRC 2/5 to 4/5. Early postoperative MRI demonstrated interval removal of the herniated fragment with satisfactory decompression of the right L5 nerve root (Figure 4A-4B). The patient was mobilized on postoperative day 1 and discharged the following day without complications. At two-week follow-up, the patient had a normal gait and MRC 5/5 strength on bedside examination.

## Discussion

This case illustrates the technical feasibility of full endoscopic interlaminar lumbar discectomy in a young adult presenting with L4-L5 disc herniation and foot drop, with favorable early clinical improvement after urgent decompression. The principal message of this report is not that full endoscopic interlaminar discectomy is superior to conventional techniques in patients with motor deficit, but that, in a carefully selected patient, urgent decompression through an endoscopic interlaminar corridor was achievable and was followed by marked early motor improvement.

Recent evidence has underscored the importance of timely surgical decompression in patients with foot drop secondary to lumbar disc herniation. Prospective data indicate that intervention within 72 hours in moderate to severe deficits may increase the likelihood of complete neurologic recovery [5]. Furthermore, a 2025 individual patient data meta-analysis demonstrated that patients treated surgically within six weeks of symptom onset have significantly better motor recovery outcomes, and that preoperative strength as well as early postoperative gains strongly predict final functional status [6]. The favorable postoperative course observed in our patient is consistent with the literature. However, this case should primarily be interpreted in the context of timely decompression, and no causal inference can be made regarding the relative contribution of surgical timing versus the specific endoscopic technique.

FELD can be performed via transforaminal or interlaminar approaches. The interlaminar approach is particularly suitable at the L4-L5 and L5-S1 levels, where the interlaminar window is relatively wide and the iliac wing or facet orientation may limit the transforaminal corridor [3]. Choi et al. reported favorable outcomes with percutaneous endoscopic interlaminar discectomy at L5-S1 [3], and Song et al. and Cheng et al. likewise demonstrated that PEID can be effectively applied to selected L5-S1 herniations, including large and calcified lesions [7,8]. Although most published series have focused on L5-S1 disease, the same anatomical principles may also apply at L4-L5 in appropriately selected patients. In the present case, the right paracentral L4-L5 herniation with inferior migration and favorable interlaminar anatomy made FELD-IL a reasonable minimally invasive option for neural decompression while preserving the posterior elements [9].

Numerous systematic reviews and meta-analyses have compared endoscopic lumbar discectomy with microdiscectomy and other minimally invasive techniques. In general, percutaneous and fully endoscopic discectomy, when performed by experienced surgeons, provide clinical outcomes comparable to conventional techniques while offering potential advantages such as reduced blood loss, shorter hospital stay, and limited soft tissue disruption [1,10,11]. Phan et al. reported outcomes at least comparable to microscopic and open discectomy, with a lower potential for complications [1]. Shi et al. found that percutaneous endoscopic lumbar discectomy was associated with shorter hospital stay, less blood loss, and similar or better functional scores compared with open microdiscectomy [11]. Latka et al. suggested that FELD may be considered a mature alternative to microdiscectomy in appropriately selected cases [12]. Nevertheless, these studies largely address lumbar disc surgery in general rather than acute motor deficit specifically, and they should not be interpreted as direct evidence that the endoscopic approach itself improves neurologic recovery in foot drop.

From a technical standpoint, the use of a small working cannula and limited soft tissue dissection may help preserve posterior stabilizing structures [4,13]. In addition, the interlaminar route offers direct access to the lateral recess and axillary region of the traversing nerve root, which may be advantageous in selected paracentral herniations at L4-L5 and L5-S1 [9]. However, these theoretical and practical advantages should be interpreted cautiously in the setting of a single case report. The present case demonstrates feasibility and a favorable early result, but it does not establish superiority over microscopic or open decompression.

Despite its potential advantages, FELD-IL also has important limitations. The learning curve is steep, and higher complication rates have been reported during the initial phase of surgical experience [14]. The working corridor is narrow, which may pose challenges in cases of large central herniations, severe spinal stenosis, or complex anatomical configurations [15]. Procedure-specific risks such as nerve root irritation, dural tears, dysesthesia, or incomplete decompression may occur, particularly in migrated or recurrent herniations [11]. Yang et al. reported that the overall complication rate of FELD is lower compared with open discectomy or microdiscectomy [10], whereas Bombieri et al. emphasized the importance of proper indication, meticulous technique, and awareness of endoscopy-specific pitfalls to prevent major complications [15]. Accordingly, careful patient selection, familiarity with interlaminar anatomy, and adequate endoscopic experience remain essential.

The role of full endoscopic lumbar discectomy in patients with acute motor deficit remains an area of ongoing interest. Our case suggests that FELD-IL may be a feasible option in carefully selected patients who require urgent decompression. However, it is equally plausible that the favorable early recovery observed here was driven primarily by the timing of decompression rather than by the endoscopic approach itself. In addition, this patient may have represented a particularly favorable anatomical case for an interlaminar endoscopic route. For these reasons, broader claims regarding the value of FELD-IL in acute motor deficit should be avoided until stronger comparative evidence becomes available.

Several limitations should be emphasized. First, this is a single case report, and the findings cannot be generalized to all patients with lumbar disc herniation presenting with foot drop. Second, the follow-up period was relatively short; therefore, longer-term neurological durability, recurrence, residual pain, return to work, and patient-reported outcomes could not be assessed. Third, no comparative data are available to determine whether the same outcome would have been achieved with microscopic or open decompression. Finally, because this was a favorable technical and anatomical scenario, the reproducibility of the same result in more complex cases remains uncertain. Larger comparative studies with longer follow-up are needed to better define the role of full endoscopic interlaminar lumbar discectomy in the management of lumbar disc herniation associated with motor deficit.

## Conclusions

In this selected patient, urgent full endoscopic interlaminar lumbar discectomy at L4-L5 was technically feasible and was followed by favorable early motor improvement. This case supports the potential applicability of the interlaminar endoscopic approach in carefully selected patients with lumbar disc herniation and foot drop, but it does not establish superiority, safety, or effectiveness relative to other decompressive techniques.
